# Disentangling etiologies of CNS infections in Singapore using multiple correspondence analysis and random forest

**DOI:** 10.1038/s41598-020-75088-4

**Published:** 2020-10-26

**Authors:** Raphaël M. Zellweger, Sophie Yacoub, Yvonne F. Z. Chan, Derek Soon, Humaira Shafi, Say Tat Ooi, Monica Chan, Leslie Jacobson, October M. Sessions, Angela Vincent, Jenny Guek Hong Low, Eng Eong Ooi, Linfa Wang, Limin Wijaya, Kevin Tan, the Singapore Neurologic Infections Program (SNIP)

**Affiliations:** 1 Emerging Infectious Diseases Program, Duke-NUS Medical School, Singapore, Singapore; 2 Viral Research and Experimental Medicine Center, SingHealth/Duke-NUS, Singapore, Singapore; 3 Antimicrobial Resistance Interdisciplinary Research Group, Singapore-MIT Alliance in Research and Technology, Singapore, Singapore; 4 Department of Infectious Diseases, Singapore General Hospital, Singapore, Singapore; 5 Department of Neurology, National University Hospital, Singapore, Singapore; 6 Department of General Medicine, Changi General Hospital, Singapore, Singapore; 7 Department of Medicine, Khoo Teck Puat Hospital, Singapore, Singapore; 8 Infectious Diseases Department, Tan Tock Seng Hospital, Singapore, Singapore; 9 Nuffield Department of Clinical Neurosciences, John Radcliffe Hospital, University of Oxford, Oxford, UK; 10 Saw Swee Hock School of Public Health, National University of Singapore, Singapore, Singapore; 11 Department of Pharmacy, National University of Singapore, Singapore, Singapore; 12 Department of Microbiology and Immunology, National University of Singapore, Singapore, Singapore; 13 Department of Neurology, National Neuroscience Institute, 11 Jalan Tan Tock Seng, Singapore, 308433 Singapore

**Keywords:** Infectious diseases, Neurological disorders, Neurological disorders, Computational models

## Abstract

Central nervous system (CNS) infections cause substantial morbidity and mortality worldwide, with mounting concern about new and emerging neurologic infections. Stratifying etiologies based on initial clinical and laboratory data would facilitate etiology-based treatment rather than relying on empirical treatment. Here, we report the epidemiology and clinical outcomes of patients with CNS infections from a prospective surveillance study that took place between 2013 and 2016 in Singapore. Using multiple correspondence analysis and random forest, we analyzed the link between clinical presentation, laboratory results, outcome and etiology. Of 199 patients, etiology was identified as infectious in 110 (55.3%, 95%-CI 48.3–62.0), immune-mediated in 10 (5.0%, 95%-CI 2.8–9.0), and unknown in 79 patients (39.7%, 95%-CI 33.2–46.6). The initial presenting clinical features were associated with the prognosis at 2 weeks, while laboratory-related parameters were related to the etiology of CNS disease. The parameters measured were helpful to stratify etiologies in broad categories, but were not able to discriminate completely between all the etiologies. Our results suggest that while prognosis of CNS is clearly related to the initial clinical presentation, pinpointing etiology remains challenging. Bio-computational methods which identify patterns in complex datasets may help to supplement CNS infection diagnostic and prognostic decisions.

## Introduction

Infections of the central nervous system (CNS) cause substantial morbidity and mortality worldwide^1^. They can be caused by bacteria, viruses, fungi, protozoa and parasites, but often the etiology remains unknown^2^. Patients with CNS infections may present with fever, headache, photophobia, and/or neck stiffness, seizures, altered consciousness and/or focal neurological signs or a combination of these features^3^. Encephalitis, most commonly of viral etiology, is associated with poor outcomes^4^. In a subset of cases, the presence of autoantibodies to cell-surface neuronal or glial proteins suggest an autoimmune etiology^2,5^.

Concern is growing about novel neurologic infections^6^, due to the emergence of new pathogens, the spread of existing pathogens to new regions as a consequence of globalization, climate change, increased virulence of existing pathogens and increasing numbers of immunocompromised patients^7–9^. This is particularly true for Southeast Asia, a focal point for zoonotic and vector-borne diseases emergence^10,11^. Singapore, a global transit hub, has recently experienced outbreaks of CNS infections of public health importance, including encephalitis from Nipah virus^12,13^, H1N1 influenza with neurological complications^14,15^ and meningoencephalitis from Group B *Streptococcal* infection^16,17^. However, etiological data of CNS infections in Singapore remain limited. A better understanding of CNS infection epidemiology would improve clinical management and public health.

The diagnosis of CNS infection is notoriously difficult, and early treatment is often empirical. Timely identification of the etiologic agent is crucial to optimize clinical care, as disease outcome often depends on tailoring treatment for the infectious agent^18^. This challenge is compounded by (1) the limited accessibility of the tissue where pathogen replication occurs, (2) the absence, in most clinical laboratories, of sensitive methods for molecular and serological detection of infection and (3) the lack of consensus on case definitions and standardized diagnostic approaches^19^. Categorizing patients into the types of CNS infections and postulating stratified etiologies based on initial clinical presentation and laboratory results would help rationalize early investigations and target treatments for the most likely etiologies.

Here, we report the epidemiology and clinical outcomes of patients with CNS infections from the Singapore Neurologic Infections Program (SNIP), a prospective surveillance study. We used multiple correspondence analysis (MCA) and random forest (RF) to uncover patterns in our complex dataset^20,21^ and explore the relationship between initial clinical presentation, laboratory results, outcome at 2 weeks, and the etiology of CNS infections.

## Results

### Etiology, demographics and epidemiology

**Figure 1:**
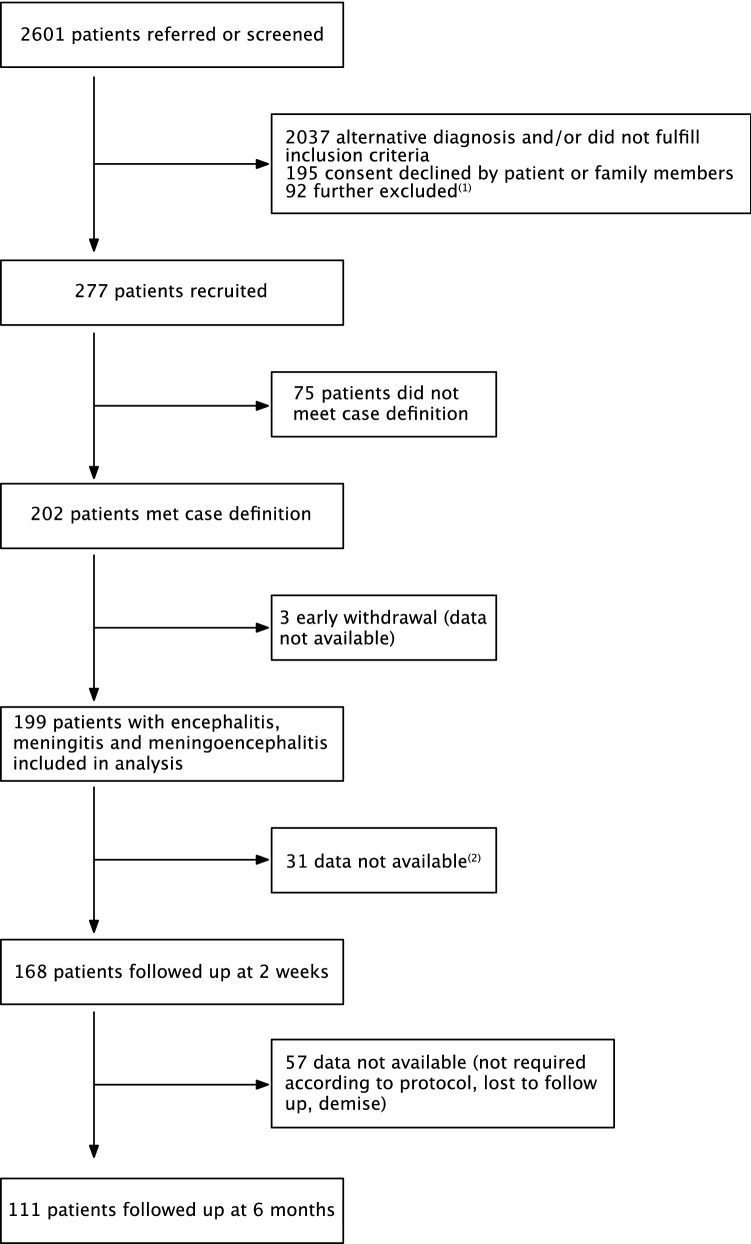
Study schematic. *Notes*: ^(1)^ These patients might or might not have fulfilled the study criteria; those who were missed either died or were not able to take consent because no legally acceptable representative was available or they were transferred out of hospital before taking consent or primary team doctors were not agreeable to recruit patients who are in serious condition or prisoners. ^(2)^ Patients may have been recruited on the discharge date or overlooked or patient withdrew from the study or declined to be followed up upon recruitment or demise.

In this study, 2061 patients were screened; 277 were recruited and 199 were included in the analysis (Fig. 1). An infectious agent was identified in 110/199 patients (55.3%, 95%-CI 48.3–62.0), an immune-mediated etiology in 10/199 (5.0%, 95%-CI 2.8–9.0), and 79/199 patients (39.7%, 95%-CI 33.2–46.6) had unknown etiology. Among the 110 cases with an infectious etiology, bacteria (excluding tuberculosis, TB) was the most common (50/110, 45.5%, 95%-CI 36.5–54.8), followed by virus (33/110, 30.0%, 95%-CI 22.2–39.1), TB (22/110, 20.0%, 95%-CI 13.6–28.4) and fungus (5/110, 4.5%, 95%-CI 2.0–10.2) (Table 1). The most common specific infectious cause was *Mycobacterium tuberculosis* (22/199, 11.1%, 95%-CI 7.4–16.1), followed by *Group B Streptococcus* (17/199, 8.5%, 95%-CI 5.4–13.3), *Treponema pallidum* (13/199, 6.5%, 95%-CI 3.9–10.9), varicella zoster virus (VZV) (12/199, 6.0%, 95%-CI 3.5–10.2), *Streptococcus pneumoniae* (11/199, 5.5%, 95%-CI 3.1–9.6) and herpes simplex virus (HSV) (10/199, 5.0%, 95%-CI 2.8–9.0). The demographic characteristics of the patients were not significantly different across etiologies, except for the presence of comorbidities and the HIV status (Supplementary Table 1). In the 14 HIV-positive patients, *Treponema pallidum* was the most common etiology, followed by cytomegalovirus (CMV) and *Cryptococcus neoformans* (Supplementary Table 2). Cerebrospinal fluid (CSF) results are summarized in Supplementary Table 3.

**Table 1:** Distribution of infectious etiologic agents causing CNS infections.

Etiology	n/N	% (95%-CI)
**Bacterial (N = 50)**		
Group B *Streptococcus*	17/50	34.0 (22.4–47.8)
*Treponema pallidum*	13/50	26.0 (15.9–39.6)
*Streptococcus pneumoniae*	11/50	22.0 (12.8–35.2)
*Klebsiella pneumoniae*	3/50	6.0 (2.1–16.2)
*Neisseria meningitidis*	2/50	4.0 (1.1–13.5)
*Haemophilus influenzae*	1/50	2.0 (0.4–10.5)
*Mycoplasma pneumoniae*	1/50	2.0 (0.4–10.5)
*Streptococcus galloly ssp pasteurianus*	1/50	2.0 (0.4–10.5)
*Streptococcus bovis*	1/50	2.0 (0.4–10.5)
**Viral (N = 33)**		
Varicella zoster virus	12/33	36.4 (22.2–53.4)
Herpes simplex virus	10/33	30.3 (17.4–47.3)
Enterovirus	5/33	15.2 (6.7–30.9)
Cytomegalovirus	2/33	6.1 (1.7–19.6)
Dengue virus	2/33	6.1 (1.7–19.6)
Epstein-Barr virus	2/33	6.1 (1.7–19.6)
**Tuberculosis (N = 22)**		
*Mycobacterium tuberculosis*	22/22	100
**Fungal (N = 5)**		
*Cryptococcus neoformans*	5/5	100

**Table 2:** Univariable regression analysis for a poor mRS outcome at 2 weeks.

Variable	OR (95% CI)	p-value
Age > 65	**6.8 (2.8–18.0)**	** < 0.001**
Female	1.5 (0.8–3.0)	0.232
Immunocompromised^(a)^	**2.6 (1.2–5.5)**	**0.015**
HIV positive	1.2 (0.2–4.6)	0.842
Altered mental status	**34.4 (12.0–125.2)**	** < 0.001**
Facial focal neurological signs	**9.0 (4.2–19.9)**	** < 0.001**
Muscle weakness	**28.5 (11.0–85.5)**	** < 0.001**
CSF protein abnormal^(b)^	1.5 (0.7–3.4)	0.344
CSF to blood glucose ratio low^(c)^	1.8 (0.8–4.8)	0.186
Neck stiffness	**2.3 (1.1–5.1)**	**0.035**
Poor mRS score at enrolment	**128.0 (40.1–582.3)**	** < 0.001**
Low sodium^(d)^	1.2 (0.6–2.4)	0.619
Abnormal movement	**9.3 (2.1–63.9)**	**0.007**
High CSF pressure	0.5 (0.2–1.2)	0.148
High WBC count^(e)^	1.5 (0.8–3.0)	0.237
Low WBC count^(e)^	0.5 (0.03–3.4)	0.560
% neutrophils in CSF > 80%	1.1(0.23–4.2)	0.886
CSF white blood cells count 5–200^(f)^	0.73 (0.24–2.34)	0.576
CSF white blood cells count > 200^(f)^	0.46 (0.14–1.6)	0.212

^(a)^Defined as history of diabetes, history of liver disease, history of renal disease, history of bone marrow transplant, history of solid organ transplant, or history of steroid use.

^(b)^Compared to normal protein level of 10–40 mg/dl.

^(c)^Defined as < 0.6.

^(d)^Defined as < 135 mEq/L.

^(e)^Compared to reference normal range of 3.5–11 × 10^6^/mL.

^(f)^Compared to reference of CSF white cells count ≤ 4/ul.

Of the 10 immune-mediated cases, 7 were diagnosed locally based on clinico-serological data: 3 N-methyl-D-aspartate receptor (NMDAR) encephalitis, and 1 each of voltage-gated potassium channel (VGKC) complex encephalitis, glutamic acid decarboxylase (GAD) encephalitis, acute disseminated encephalomyelitis (ADEM) and Bickerstaff encephalitis. Results from the Oxford Neuroimmunology laboratory identified additional 3 cases with NMDAR encephalitis.

**Figure 2:**
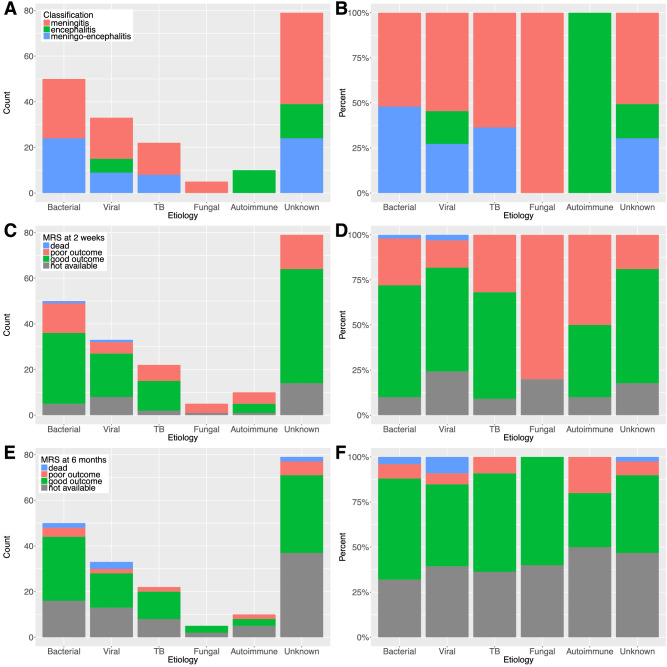
Distribution of diagnosis (**A**,**B**) and clinical outcomes (**C**–**F**) stratified by etiology. Absolute counts (**A**) and percentages (**B**) of cases with diagnoses of meningitis, encephalitis or meningo-encephalitis, stratified by etiology. Absolute counts and percentages of cases with different clinical outcomes at 2 weeks (**C**,**D**) and 6 months (**E**,**F**), stratified by etiology.

Figure 2A,B show the absolute counts and percentages of patients with meningitis, encephalitis or meningoencephalitis stratified by etiology. Bacterial and TB infections caused both meningitis and meningoencephalitis; fungal infections caused only meningitis. Interestingly, viral infections and the unknown etiology group had similar proportions of all 3 syndromes.

The counts and percentages of good or poor outcomes (as measured by the modified Rankin Scale (mRS) score ≤ 2 or ≥ 3, respectively) at two weeks and six months, stratified by etiology, are shown in Fig. 2C–F. At 2 weeks, fungal infections caused greatest morbidity, with poor outcomes in more than 75% of cases, followed by autoimmune etiology, TB and bacterial infections. Similarly, viral infections and the unknown etiology group had similar proportions of good and poor outcomes.

With univariable regression analysis, the following variables measured at enrolment were significantly associated with a poor outcome at two weeks: age over 65 years old, immunocompromised, altered mental status, facial focal neurological signs, muscle weakness, neck stiffness, abnormal movements and mRS score of 3–5 (Table 2).

### Multiple correspondence analysis

**Figure 3:**
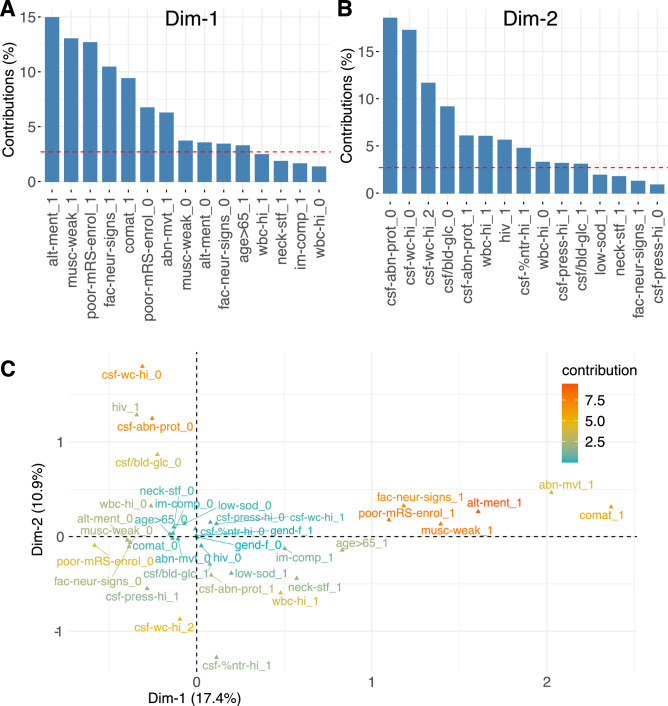
Variables of the MCA—contribution to the first 2 dimensions and correlation. Contribution of variables (expressed in %) to the first (**A**) and second (**B**) dimension of the MCA. The dotted red line denotes the average value expected if all variables contributed equally to the dimensions. Presence or absence of a variable is denoted by 1 or 0 after the name, except for the CSF white cell count where 0, 1 and 2 denote ≤ 4, 5–200 and > 200 cells/ul, respectively. Dimension 1 is related to the initial clinical presentation, dimension 2 is related to laboratory measurements (see text for details). On the correlation plot of the variables (**C**), variable contribution to the dimensions of the MCA is indicated in color and distance is inversely proportional to the correlation between variables. Variable abbreviations: "poor-mRS-enrol": poor mRS score at enrolment, "im-comp": immunocompromised, "hiv": HIV-status, "alt-ment": altered mental status, "comat": comatose, "neck-stf": neck stiffness, "fac-neur-signs": facial focal neurological signs, "musc-weak": muscle weakness, "abn-mvt": abnormal movements, "csf-abn-prot": abnormal level of protein in the CSF, "csf/bld-glc" low CSF to blood glucose ratio, "csf-wc-hi": elevated CSF white cell count, "csf-%ntr-hi": elevated percentage of neutrophils in the CSF, "gend-f": female gender, "wbc-hi": elevated white blood cells, "low-sod": low sodium, "csf-press-hi": elevated opening pressure, "age > 65": age over 65.

For multivariable analysis, multiple correspondence analysis (MCA) was performed. Dimension 1 was composed of variables that pertain to the clinical presentation of the patient, such as altered mental status, poor mRS score at enrolment, muscle weakness, facial focal neurological signs and comatose state; this dimension captured 17.4% of the variance in the data points. Dimension 2 was composed of variables related to laboratory measurements, such as abnormal CSF protein concentration, CSF white cell count, CSF to blood glucose ratio, blood white blood cell count as well as HIV status (Fig. 3A,B); this dimension captured 10.9% of the variance in the data points. The correlation between the variables of the MCA is presented in Fig. 3C. The closer two variables are located on the plane, the more correlated they are.

**Figure 4:**
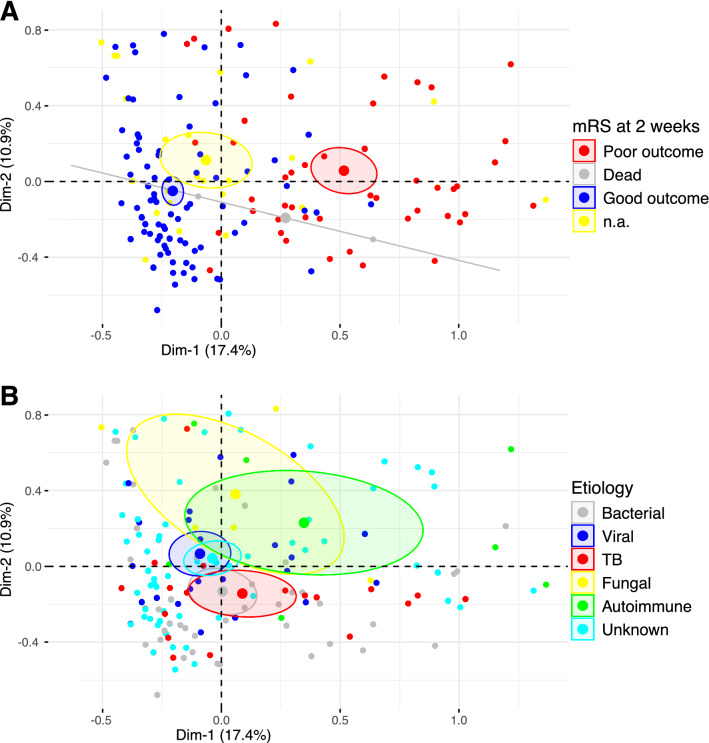
Individuals on the MCA plane, colored by clinical outcome (**A**) or etiology (**B**). Representation of the individuals on the plane defined by dimensions 1 and 2 of the MCA, stratified by mRS score at 2 weeks (**A**) or by etiology (**B**). The larger dot and the ellipse represent the barycenter of the cloud of points, and the 95% confidence interval of the barycenter. Dimension 1 is related to the initial clinical presentation, dimension 2 is related to laboratory measurements (see text for details).

The distribution of the subjects on the MCA plane was analyzed by clinical outcome at two weeks (Fig. 4A). Cases with good outcome at two weeks were mainly grouped on the left-hand side while cases with poor outcomes were located on the right. As dimension 1 mainly relates to the clinical presentation at enrolment, this suggests that clinical presentation at enrolment is indicative of clinical outcome at two weeks.

Next, the distribution of the subjects on the MCA plane was analyzed by etiology (Fig. 4B). Cases with TB or bacterial etiologies occupied the lower section of the graph. Cases with fungal or autoimmune etiology occupied the upper section of the graph. The point distribution suggests that patients with CSF white cell count > 200 and/or neutrophils percentage > 80% were more likely to be of bacterial or TB etiology. Absence of white cells in the CSF, absence of abnormal protein level in the CSF, or positive HIV-status were suggestive of fungal or autoimmune etiology.

### Random forest

**Figure 5:**
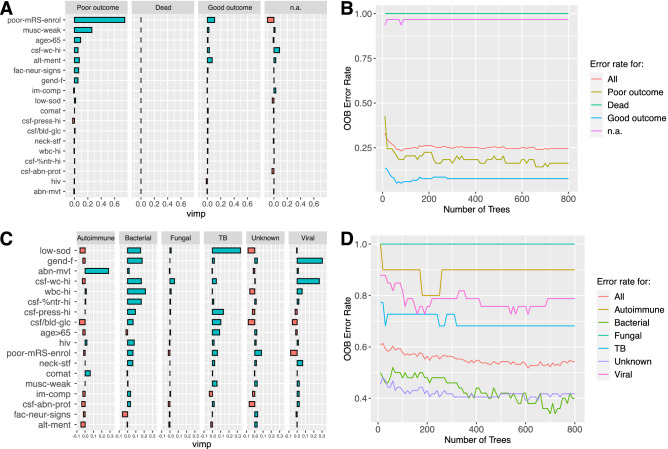
Random forest (RF) analysis for clinical outcome at 2 weeks (**A**,**B**) and etiology (**C**,**D**). A random forest analysis was performed with the clinical outcome at 2 weeks as classifier (**A**,**B**) or the etiology as classifier (**C**,**D**). (**A**) Variable importance for each of the possible outcomes (poor outcome, dead, good outcome, n.a.). (**B**) Error rate (based on out-of-the-bag cross-validation) as a function of the number of trees generated. (**C**) Variable importance for each of the possible etiologies (autoimmune, bacterial, fungal, TB, unknown, viral). (**D**) Error rate (based on out-of-the-bag cross-validation) as a function of the number of trees generated.

Random Forest (RF) analysis was performed with the clinical outcome at 2 weeks or the etiology as classifier. The importance of the variables for the different outcomes at 2 weeks (good, poor, dead or n.a.) is presented in Fig. 5A. The error rate as function of the number of trees generated is presented in Fig. 5B. The overall error rate was close to 25%, but the error rate for classification of good and poor outcome was much lower. Collectively, the RF analysis suggests that a poor mRS score at enrolment was strongly associated with a poor outcome at 2 weeks. The importance of the variables for the different etiologies is presented in Fig. 5C. The high error rate (overall above 50%, Fig. 5D) suggests that the variables available did not reliably discriminate between all the etiologies. This is not surprising considering the high degree of overlap observed in the MCA between TB and bacterial, viral and unknown, and fungal and autoimmune.

## Discussion

The identification of CNS infection etiology remains a major challenge in all health systems throughout the world due to limited access to the infection site, delay in the timely collection of relevant clinical samples, as well as limitations of current diagnostic tests. The practice of early empirical antimicrobial treatment upon suspicion of CNS infection is important for optimal patient care, but may confound the diagnostic process. In this study, we made an effort to prospectively and systematically define the etiology of CNS infections in Singapore. We observed that more than half (55.3%) of the CNS infections had an infectious etiology identifiable with diagnostic tests currently available in clinical laboratories. CNS infections were most frequently bacterial, followed by viral, TB and fungal. Around two-fifths of patients (39.7%) remained without a definitive etiology, slightly lower than the 48% and 52% recently reported in Vietnam^22^ and Thailand^23^, respectively.

In our cohort, TB was the most frequent specific etiology for CNS infection. The incidence of TB has increased in Singapore in recent years, possibly due to factors such as the influx of immigrants from highly endemic countries, an ageing population and high prevalence of diabetes mellitus, a risk factor for TB^24^. Our data suggests that, similar to neighboring Malaysia^25^, TB is a common etiologic agent for CNS infections.

The high number of Group B *Streptococcus* detected was linked to the epidemic that occurred in Singapore in 2015, which was associated with consumption of raw fish^26^. VZV and HSV were the most common viral etiologies identified. No Japanese Encephalitis Virus (JEV) was detected in our cohort, possibly because of the low prevalence of JEV in Singapore since the elimination of pig farming^27^. In the rest of Asia and globally, JEV remains a common cause of viral encephalitis, as for example in Vietnam^22^. In Taiwan, HSV and VZV were the most frequent cause of encephalitis in a hospital-based study^28^. In Europe, the most frequent etiologies for CNS infections were *Streptococcus pneumoniae* (8%), *Mycobacterium tuberculosis* (5.9%), followed by VZV and Listeria in the elderly^1^.

In our cohort, the most frequent etiology for CNS infection in HIV-positive patients was *Treponema pallidum* (6/14), with *Cryptococcus neoformans* accounting for only two cases. This contrasts with studies in the United States, Europe and Uganda, where *Cryptococcus neoformans* was the leading cause of meningitis in HIV patients^1,29,30^. Surprisingly, despite a high overall proportion of TB meningitis in the SNIP cohort, TB was not detected in HIV-positive patients.

Immune-mediated causes of encephalitis form a significant proportion of cases of previous unknown cause. In our cohort, 10 cases (5.0%) were identified through a combination of tests done as part of clinical evaluation and a systematic screen of autoantibodies in a research laboratory. A prospective study from the UK identified 20% of cases with an immune-mediated cause, including ADEM, NMDAR encephalitis and VGKC complex encephalitis^31^; another from Thailand, found 24% of patients with encephalitis were associated with immune encephalitis^23^. A recent retrospective cohort study in the United States also identified 20% with an autoimmune cause^32^; another from Vietnam found 9.1% had NMDAR encephalitis^33^. A systematic approach to autoantibody testing in the assessment of acute encephalitis patients may be required as early and accurate diagnosis of immune-mediated etiology is important for appropriate immunotherapy rather than empirical administration of antimicrobials.

A second major issue in the management of CNS infection is prognostication and resource allocation to manage patients. While viral meningitis generally has a good prognosis^34^, acute bacterial meningitis or viral encephalitis tend to be more severe and can be fatal^18^. Therefore, recognizing the clinical syndrome and the etiology is crucial to optimize clinical care and improve patient management. In this study, we explored the use of bio-computational methods to aid in the diagnosis and prognostication of patients with suspected CNS infections. Using MCA, we showed that some of the features at the initial clinical presentation, such as altered mental status, poor mRS score at enrolment, muscle weakness, focal facial neurological signs, abnormal movements and comatose state correlated with a poor outcome at two weeks. RF analysis suggested that poor mRS score at enrolment and muscle weakness were the two factors most strongly associated with a poor outcome at 2 weeks, followed by age > 65 years, elevated leukocytes in the CSF, altered mental status and focal facial neurological signs. Being aware of these features early in the disease course allows clinicians to decide which patients they need to be more vigilant with and devote resources to manage them.

To identify possible etiologies, MCA showed that laboratory-related variables were important. Normal CSF protein levels, low CSF white cell count (≤ 4) and/or an HIV-positive status was suggestive of fungal or autoimmune etiology; very high CSF white cell count (> 200 cells/ul) and/or high white blood cells (> 11 × 10^6^ cells/mL) was associated with TB or bacterial etiology. However, RF analysis could not reliably discriminate between all etiologies based on the available predictors. This is not surprising as there was considerable overlap observed in the MCA between bacterial and TB, fungal and autoimmune, and viral and unknown. It suggests that the predictors available could hint at a bacterial/TB or viral/unknown or auto-immune, but could not further differentiate between greatly overlapping groups (Fig. 3B). Nevertheless, this initial stratification may still be helpful to guide early clinical care. Interestingly, cases with viral and unknown etiologies were mostly overlapping, and therefore mostly similar with respect to the variables measured in our study. This suggests that cases without confirmed etiology may have been viral. This hypothesis is also supported by our finding that the distribution of meningitis, encephalitis and meningo-encephalitis was almost identical between CNS of viral and unknown etiologies.

Identifying other clinical, laboratory and investigation parameters for analysis may allow refinement of the bio-computational methods. The methods may also be attempted with larger cohorts of CNS infection patients for validation. Nonetheless, our data reaffirms that definitive diagnostic tests to reliably discriminate between infectious and non-infectious etiologies for CNS infections are urgently needed.

The strengths of our study were its prospective design and multidisciplinary recruitment from all the major public hospitals in Singapore, which provides medical care to approximately 70–80% of the population. The patients were enrolled by acute care hospitals and therefore are likely to be representative of the range of clinical presentations and causes encountered in Singapore. A limitation of the study was the inclusion of only adult cases. In addition, the clinical management of the cases was left to the treating physicians, hence the investigations and treatments would have varied. From an analysis perspective, MCA is a powerful technique to detect and represent patterns in large datasets. However, it does not formally prove associations between measured variables and outcomes. RF, on the other hand, outputs a ranking of the relative importance of variables in classifying outcomes, but does not quantify the absolute contribution of each variable in determining the outcome.

In conclusion, **t**his prospective study described the epidemiology of CNS infections in Singapore, and highlighted a surprisingly high proportion of TB meningitis in our cohort. Our analysis using MCA and RF also suggests that initial clinical features at presentation informs prognosis at two weeks, while laboratory parameters may aid stratification to various etiologies and guide early clinical care. Our study supports the utility of bio-computational algorithms to analyze the wealth of data routinely collected in most clinical settings. However, the parameters measured in clinical practice not being able to discriminate completely between the etiologies demonstrates the urgent need to develop better diagnostic tests to enhance the current diagnostic toolbox and accurately determine the etiology of CNS infection.

## Methods

### Study design and ethics

We performed a prospective surveillance cohort study in five adult general hospitals (Singapore General Hospital, National University Health System, Changi General Hospital, Tan Tock Seng Hospital, and Khoo Teck Puat Hospital) and one national tertiary center (National Neuroscience Institute) between August 2013 and December 2016. The study was approved by the SingHealth Centralized Institutional Review Board (CIRB Ref no. 2013/374/F) and NHG Domain Specific Review Boards (2013/01259) and all experiments were performed in accordance with relevant guidelines and regulations. Informed consent was obtained from patients or from the next of kin for patients who were unconscious or incapable of exercising rational judgment.

### Inclusion and exclusion criteria

Inpatients were enrolled if they met the study inclusion criteria: (1) clinical suspicion for CNS infection or (2) any two of the following symptoms—(a) fever or history of fever (≥ 38 °C) during the presenting illness, (b) new onset of seizure, (c) focal neurological deficits, (d) CSF pleocytosis (> 4 white blood cells/uL), (e) abnormal neuroimaging suggestive of CNS infection, (f) abnormal electroencephalogram (EEG) suggestive of CNS infection, (g) depressed or altered level of consciousness, or (3) no alternative etiology for acute paralysis identified. Patients were excluded: (1) if they had existing indwelling ventricular device or (2) if they or their relatives did not provide written informed consent.

### Procedures

Patients were enrolled and followed up at 2 weeks or at discharge, whichever was earlier, and at six months (by questionnaire or during a coincident hospital visit). Sera and CSF were collected during acute disease at the time of hospitalization and, when possible, convalescent sera were collected 2–4 weeks later. This study did not interfere with the clinical management of the patients. The following data were collected: demographics, presenting symptoms, past medical history, neuroimaging and neurophysiology tests, laboratory investigations including CSF results and all therapeutic interventions. The modified Rankin Scale (mRS) was used to measure the degree of disability and dependence in daily activities at enrolment, two weeks (or discharge) and six months. An mRS scores of 0–2 denoted a good outcome, while a score of 3–5 denoted a poor outcome.

### Case definition

The clinical diagnosis of patients was classified as encephalitis, meningitis, or meningoencephalitis. No cases of encephalomyelitis were found. Patients with encephalitis, defined as inflammation of the brain parenchyma associated with neurologic dysfunction, had altered mental status (decreased level of consciousness, lethargy, personality change, and unusual behavior), seizures, focal neurological signs, and/or fever. Patients with meningitis, defined as inflammation of the meninges, had fever, headache, photophobia, and neck stiffness. Meningoencephalitis patients had a combination of the above features.

Based on laboratory results, etiologies were classified as bacterial, viral, tuberculosis (TB), fungal or autoimmune. Infectious etiologies were classified as “confirmed”: (1) infectious agent detected in CSF by polymerase chain reaction (PCR), serological or molecular testing and (2) clinical presentation consistent with infection, or “probable”: (1) infectious agent detected extra-cranially (e.g. blood) by PCR, serological or molecular testing or (2) clinical presentation consistent with infection and patient responds to specific antimicrobial treatment. Both confirmed and probable cases are included in this analysis.

The diagnosis of autoimmune encephalitis was based on (1) conventional clinical neurological assessment and standard diagnostic tests, (2) absence of identification of an infectious agent, and (3) presence of N-methyl-D-aspartate receptor (NMDAR), voltage-gated potassium channel (VGKC) complex, contactin-associated protein like 2 (CASPR2), leucine-rich glioma inactivated 1 (LGI1), gamma-aminobutyric acid _A_ receptor (GABAAR) or glutamic acid decarboxylase (GAD) autoantibodies in the serum and/or CSF^5^. Autoantibody tests were performed in the Singapore hospitals’ clinical laboratories at the managing clinicians’ discretion. All sera samples were subsequently tested systematically for the above autoantibodies in the Oxford Neuroimmunology laboratory (Oxford University Hospitals, United Kingdom). Live cell-based assay was used for the detection of IgG antibodies binding to the NMDAR NR1/NR2b subunits, CASPR2, LGI1 and α1 and γ2 subunits of GABAAR. Binding to the cell membrane was scored by fluorescence microscopy. VGKC complex antibodies was measured using a radioimmunoprecipitation assay of VGKC complex proteins labelled with ^125^I-α-dendrotoxin and precipitated with patient serum samples^35^.

### Statistical analysis

Statistical analysis was performed using R version 3.4.1^36^ and R-packages ggplot2^37^, FactoMineR^38^ for the multiple correspondence analysis (MCA) and missMDA^39^ for imputation of missing values. For the random forest (RF) analysis, packages randomForestSRC^40^ and ggRandomForests^41^ were used. For univariable analysis, the association between explanatory variables and poor outcome at two weeks (mRS ≥ 3) was assessed by Fisher’s exact test and expressed as odds ratio (OR).

MCA is a non-supervised exploratory principal component method that can be performed on datasets containing qualitative variables. MCA does not require an a priori knowledge of the correlation between variables, nor does it make any assumption about their distribution. For these reasons, MCA is particularly useful to analyze large epidemiological datasets^20,21^. The goal of MCA is to simplify complex datasets by reducing the number of variables in order to uncover patterns in the data; results are represented graphically for easy interpretation. During MCA, the independent variables are grouped into a smaller number of uncorrelated dimensions (factorial axes) that describe the spread (or variance) of points. Each dimension explains a progressively decreasing percentage of the spread of the data points.

All variables used in univariable analysis were used to construct the factorial axes of the MCA. Etiology, mRS outcome at two weeks and mRS outcome at six months were outcome variables, and were excluded from the factorial axes construction. They were used to classify the individuals on the MCA plane, allowing us to explore patterns between individuals with similar outcome or etiologies. Interpretation of the results is based on the distance between the data points and their position along the dimensions. Points similar with respect to the independent variables are closer to each other. Similarly, the closer the variables are in the MCA space, the more correlated they are.

RF is a versatile supervised machine learning algorithm that can be used for classification or regression^42^. A RF consists of an ensemble of decision trees made of a random sample of the available predictor variables. In isolation, the predictive accuracy of each tree is low, but the prediction is vastly improved by growing a large ensemble of trees (a forest) and letting them “vote” for the most likely class. RF is widely used in life sciences because of its high-prediction accuracy and the fact that it outputs information on the importance of variables for the classification problem at hand. Conveniently, the RF algorithm outputs the importance of the various predictor variables for each outcome of interest. Error rate for classification can be generated using the built-in cross-validation algorithm, where each tree in the forest has its own training (bootstrap) and test (out-of-bag, OOB) data^43^. In our study, we used RF to assess the relative importance of the variables used for univariable analysis in classifying either the clinical outcome at two weeks or the etiology.

### Ethics approval

The study was approved by the SingHealth Centralized Institutional Review Board (CIRB Ref no. 2013/374/F) and NHG Domain Specific Review Board (2013/01259).

### Consent to participate

Informed consent was obtained from patients or from the next of kin for patients who were unconscious or incapable of exercising rational judgment.

## Supplementary information

Supplementary Information

## Data Availability

The datasets used and/or analyzed during the current study are available from the corresponding author on reasonable request.

## Abbreviations

ADEM: Acute disseminated encephalomyelitis
CASPR2: Contactin-associated protein 2
CNS: Central nervous system
CSF: Cerebrospinal fluid
GABAAR: Gamma-aminobutyric acid_A_ receptor
GAD: Glutamic acid decarboxylase
HIV: Human immunodeficiency virus
HSV: Herpes simplex virus
JEV: Japanese encephalitis virus
LGI1: Leucine-rich glioma inactivated 1
MCA: Multiple correspondence analysis
mRS: Modified Rankin Scale
NMDAR: *N*-Methyl-d-aspartate receptor
PCA: Principal component analysis
RF: Random forest
TB: Tuberculosis
VGKC: Voltage-gated potassium channel
VZV: Varicella zoster virus
WBC: White blood cells
