# Supplementary material for: Disentangling etiologies of CNS infections in Singapore using multiple correspondence analysis and random forest
Source: Sci Rep. 2020 Oct 26;10:18219. doi: 10.1038/s41598-020-75088-4 (PMC7588471; doi:10.1038/s41598-020-75088-4)
Supplement: Supplementary file 1 — Supplementary Information [file 41598_2020_75088_MOESM1_ESM.docx]

**Disentangling etiologies of CNS infections in Singapore using multiple correspondence analysis and random forest**

Raphaël M. Zellweger^1,2^, Sophie Yacoub^3^, Yvonne FZ Chan^4^, Derek Soon^5^, Humaira Shafi^6^, Say Tat Ooi^7^, Monica Chan^8^, Leslie Jacobson^9^, October M. Sessions^1,10,11^, Angela Vincent^9^, Jenny Guek Hong Low^1,4^, Eng Eong Ooi^1,2,3,10,12^, Linfa Wang^1^, Limin Wijaya^4,#^, Kevin Tan^13,#,*^

on behalf of the Singapore Neurologic Infections Program (SNIP)

^1^ Emerging Infectious Diseases Program, Duke-NUS Medical School, Singapore

^2^ Viral Research & Experimental Medicine Center @ SingHealth/Duke-NUS, Singapore

^3^ Singapore-MIT Alliance in Research and Technology, Antimicrobial Resistance Interdisciplinary Research Group, Singapore

^4^ Department of Infectious Diseases, Singapore General Hospital, Singapore

^5^ Department of Neurology, National University Hospital, Singapore

^6^ Department of General Medicine, Changi General Hospital, Singapore

^7^ Department of Medicine, Khoo Teck Puat Hospital, Singapore

^8^ Infectious Diseases Department, Tan Tock Seng Hospital, Singapore

^9^ Nuffield Department of Clinical Neurosciences, John Radcliffe Hospital,

University of Oxford, Oxford, UK

^10^ Saw Swee Hock School of Public Health, National University of Singapore, Singapore, Singapore.

^11^ Department of Pharmacy, National University of Singapore, Singapore

^12^ Department of Microbiology and Immunology, National University of Singapore, Singapore, Singapore

^13^ Department of Neurology, National Neuroscience Institute, Singapore

^#^ Both authors contributed equally to this work

*** Corresponding author:** Kevin Tan, Department of Neurology, National Neuroscience Institute, 11 Jalan Tan Tock Seng, Singapore 308433; Tel: 65-63577171; Fax: 65-63577137; E-mail: [kevin.tan@singhealth.com.sg](mailto:kevin.tan@singhealth.com.sg); ORCID 0000-0001-5894-8839

**Keywords:**

- central nervous system infections
- autoimmune encephalitis
- tuberculosis
- multiple correspondence analysis
- random forest

|  | **Etiology** | | | | | | | | ***p*-value^(a)^** |
| --- | --- | --- | --- | --- | --- | --- | --- | --- | --- |
|  | **Total (N=199)** | | **Bacterial (N=50)** | **Viral (N=33)** | **TB (N=22)** | **Fungal (N=5)** | **Autoimmune (N=10)** | **Unknown (N=79)** |  |
| **Demographics** |  | |  |  |  |  |  |  |  |
| Age over 65, n (%) | 31 (15.6) | | 8 (16) | 6 (18.2) | 4 (18.2) | 0 (0) | 2 (20.0) | 11 (13.9) | 0.936 |
| Gender, M/F (% males) | 126/73 (63.3) | | 30/20 (60) | 19/14 (57.6) | 17/5 (77.3) | 4/1 (80) | 5/5 (50.0) | 51/28 (64.6) | 0.575 |
| **Ethnicity, n (%)** | | | | | | | | | **0.612** |
| Chinese | 145 (72.9) | | 41 (82.0) | 25 (75.8) | 15 (68.2) | 5 (100) | 4 (40.0) | 55 (69.6) |  |
| Malay | 23 (11.6) | | 4 (8.0) | 4 (12.1) | 3 (13.6) | 0 (0) | 2 (20.0) | 10 (12.7) |  |
| Indian | 16 (8.0) | | 3 (6.0) | 3 (9.1) | 3 (13.6) | 0 (0) | 2 (20.0) | 5 (6.3) |  |
| Eurasian | 1 (0.5) | | 0 (0) | 0 (0) | 0 (0) | 0 (0) | 0 (0) | 1 (1.3) |  |
| Other | 14 (7.0) | | 2 (4.0) | 1 (3.0) | 1 (4.5) | 0 (0) | 2 (20.0) | 8 (10.1) |  |
| **Comorbidities, n (%)** | | | | | | | | | |
| Any comorbidity | | 81 (40.7) | 26 (52.0) | 15 (45.5) | 10 (45.5) | 4 (80.0) | 3 (30.0) | 23 (29.1) | **0.044** |
| Diabetes | | 30 (15.1) | 10 (20.0) | 4 (12.1) | 4 (18.2) | 0 (0) | 1 (10.0) | 11 (13.9) | 0.874 |
| Moderate or severe liver  disease | | 5 (2.5) | 3 (6.0) | 1 (3.0) | 0 (0) | 0 (0) | 1 (10.0) | 0 (0) | 0.097 |
| Moderate or severe renal  disease | | 6 (3.0) | 1 (2.0) | 2 (6.1) | 0 (0) | 1 (20.0) | 0 (0) | 2 (2.5) | 0.254 |
| Any solid tumor | | 17 (8.5) | 6 (12.0) | 2 (6.1) | 2 (9.1) | 0 (0) | 1 (10.0) | 6 (7.6) | 0.901 |
| Autoimmune  disease/connective tissue  disease | | 5 (2.5) | 1 (2.0) | 1 (3.0) | 1 (4.5) | 1 (20.0) | 0 (0) | 1 (1.3) | 0.189 |
| Chronic use of steroid or  immunosuppressant  (>21days) | | 10 (5.0) | 1 (2.0) | 1 (3.0) | 3 (13.6) | 1 (20.0) | 0 (0) | 4 (5.1) | 0.176 |
| HIV | | 14 (7.0) | 6 (12.0) | 4 (12.1) | 0 (0) | 2 (40.0) | 0 (0) | 2 (2.5) | **0.012** |

**Supplementary Table 1: Demographics and comorbidities of the study population, stratified by etiology**

^(a)^ Fisher’s exact test for proportion of each variable in the different etiologies

**Supplementary Table 2: Distribution of etiologies in HIV-positive patients**

| **Etiology (N=14)** | **n/N** | **% (95%-CI)** |
| --- | --- | --- |
| *Treponema pallidum* | 6/14 | 42.9 (21.4-67.4) |
| Cytomegalovirus | 2/14 | 14.3 (4.0-39.9) |
| *Cryptococcus neoformans* | 2/14 | 14.3 (4.0-39.9) |
| Unknown | 2/14 | 14.3 (4.0-39.9) |
| Herpes simplex virus | 1/14 | 7.1 (1.3-31.5) |
| Varicella zoster virus | 1/14 | 7.1 (1.3-31.5) |

**Supplementary Table 3: CSF results in the study population**

|  | **Etiology** | | | | | | | |  |
| --- | --- | --- | --- | --- | --- | --- | --- | --- | --- |
| **Number (n) with abnormal**  **CSF investigations** | | **Total** | **Bacterial** | **Viral** | **TB** | **Fungal** | **Autoimmune** | **Unknown** | ***p*-value^(a)^** |
| **CSF/Blood glucose <0.6,** n/N^(b)^ (%) | | 90/134 (67.2) | 24/30 (80) | 9/19 (47.4) | 16/18 (88.9) | 2/2 (100) | 2/6 (33.3) | 37/59 (62.7) | **0.012** |
| **Elevated CSF white cell,** n/N (%) | |  |  |  |  |  |  |  | **0.049** |
| CSF white cells: 5-200 cells/ul | | 111/195 (56.9) | 18/47 (38.3) | 22/33 (66.7) | 15/22 (68.2) | 2/5 (40.0) | 8/10 (80.0) | 46/79 (59.0) |  |
| CSF white cells: >200 cells/ul | | 62/195 (31.8) | 25/47 (53.2) | 9/33 (27.3) | 6/22 (27.3) | 0/5 (0) | 1/10 (10.0) | 21/79 (26.9) |  |
| **Protein^(c)^,** n/N (%) | | 146/195 (74.9) | 40/47 (85.1) | 25/33 (75.8) | 20/22 (90.9) | 3/5 (60.0) | 4/6 (40.0) | 54/78 (69.2) | **0.014** |

^(a)^ Fisher’s exact test for proportion of each variable in the different etiologies

^(b)^ n= number with abnormal finding, N= total tested

^(c)^ abnormal protein level of 10-40
